# Therapy of established B16-F10 melanoma tumors by a single vaccination of CTL/T helper peptides in VacciMax^®^

**DOI:** 10.1186/1479-5876-5-20

**Published:** 2007-04-23

**Authors:** Marc Mansour, Bill Pohajdak, W Martin Kast, Antar Fuentes-Ortega, Ella Korets-Smith, Genevieve M Weir, Robert G Brown, Pirouz Daftarian

**Affiliations:** 1 ImmunoVaccine Technologies Inc., Halifax, NS, Canada; 2 Dept. of Molecular Microbiology & Immunology and Norris Comprehensive Cancer Center, University of Southern California, Los Angeles, USA; 3 Department of Microbiology & Immunology, Dalhousie University, Halifax, NS, Canada

## Abstract

**Background:**

Melanoma tumors are known to express antigens that usually induce weak immune responses of short duration. Expression of both tumor-associated antigens p53 and TRP2 by melanoma cells raises the possibility of simultaneously targeting more than one antigen in a therapeutic vaccine. In this report, we show that VacciMax^® ^(VM), a novel liposome-based vaccine delivery platform, can increase the immunogenicity of melanoma associated antigens, resulting in tumor elimination.

**Methods:**

C57BL/6 mice bearing B16-F10 melanoma tumors were vaccinated subcutaneously 6 days post tumor implantation with a mixture of synthetic peptides (modified p53: 232–240, TRP-2: 181–188 and PADRE) and CpG. Tumor growth was monitored and antigen-specific splenocyte responses were assayed by ELISPOT.

**Results:**

Vaccine formulated in VM increased the number of both TRP2- and p53-specific IFN-γ producing splenocytes following a single vaccination. Vaccine formulated without VM resulted only in enhanced IFN-γ producing splenocytes to one CTL epitopes (TRP2:180–188), suggesting that VM overcomes antigen dominance and enhances immunogenicity of multiple epitopes. Vaccination of mice bearing 6-day old B16-F10 tumors with both TRP2 and p53-peptides formulated in VM successfully eradicated tumors in all mice. A control vaccine which contained all ingredients except liposomes resulted in eradication of tumors in no more than 20% of mice.

**Conclusion:**

A single administration of VM is capable of inducing an effective CTL response to multiple tumor-associated antigens. The responses generated were able to reject 6-day old B16-F10 tumors.

## Background

Previous studies using two mouse models have demonstrated that peptide-based vaccines delivered using VacciMax^® ^(VM) can eradicate human papillomavirus (HPV) 16 induced tumors [1,2]. The aim of the present study is to use VM in a therapeutic vaccine to treat C57BL/6 mice bearing B16-F10 melanoma tumors. Eradication of both HPV 16 and B16-F10 melanoma tumors would suggest that VM could enhance various therapeutic cancer vaccines. The identification of tumor-associated antigens that are recognized by CD8^+ ^cytotoxic T lymphocytes (CTL) has led to the development of specific anti-tumor immunotherapies. Vaccine approaches for the treatment of cancer rely on the expansion and differentiation of tumor-specific CTL's, with the objective of rejecting established tumor. Peptide vaccines elicit a poor immune response and often require the use of potent adjuvants. Previous studies in our laboratory have shown that formulation of peptides in a vaccine enhancing platform, VM, can greatly enhance peptide-specific immune responses. VM is a liposome-based antigen delivery platform which we have shown to be effective in both preventing and eradicating established HPV 16-induced tumors in mouse models [1,2]. The strong and specific CTL responses generated by VM against a variety of peptide antigens suggest that VM can be used to develop therapeutic vaccines against a variety of cancers. This report examines the use of VM in a melanoma mouse model.

A number of melanoma-associated antigens (MAA) have been identified as potential targets but most induce weak immune responses of limited duration. MAAs, expressed by both normal and malignant melanocytes, are self antigens that are difficult to target in a vaccine. MAAs include melanoma-specific antigens such as MART-1, gp100 and TRP2 [3], as well as antigens such as mutated p53 found in a variety of cancers [4-11]. The melanoma tumor cell line B16-F10 used in this study expresses two MAAs of interest, namely TRP-2 and mutated p53.

In this study, VM containing TRP2 and p53 CTL epitopes was used as a therapeutic vaccine to treat C57BL/6 mice bearing B16-F10 melanoma tumors. TRP2 was selected because clinical tumor regression has been associated with TRP2-specific T cells [12-17]. p53, on the other hand, was selected because it is expressed in a variety of cancers including melanoma [5,18-23], and the ability to induce effective immune responses against p53 in a melanoma model may have implications for the treatment of other cancers.

## Methods

### Mice and cell lines

Pathogen-free C57BL/6 female mice, 6–8 weeks of age, were obtained from Charles River Laboratories (Wilmington, MA) and were housed under filter top conditions with water and food *ad libitum*. The B16-F10 melanoma cell line was obtained from American Type Culture Collection (ATCC, Manassas, VA). The melanoma cell line was cultured in Dulbecco's Modified Dulbecco's Medium (DMDM, Sigma, St. Louis, MO) supplemented with 10% heat-inactivated fetal calf serum (Hyclone), 2 mM L-glutamine (Gibco), 5 mM 2-mercaptoethanol (Gibco), penicillin and streptomycin (100 μg/ml;Gibco). Cells were incubated at 37°C/5% CO_2_.

### Peptides and oligonucleotide (ODN)

The tyrosinase-related protein peptides TRP2: 180–188 (SVYDFFVWL) and TRP2: 181–188 (VYDFFVWL), as well as the modified p53 peptide (p53: 232–240; KYICNSSCM) fused to PADRE (AKXVAAWTLKAAA, where X is cyclohexylalanine) were purchased from Dalton Chemical Laboratories Inc. (Toronto, ON, Canada). These peptides are presented by murine MHC-class I H-2K. The TRP2 and p53 peptides were stored as a 1 mg/ml stock solution containing DMSO to maintain solubility. Further dilutions for vaccine production were made using PBS.

All formulations of the vaccines contained either PADRE (AKXVAAWTLKAAA-OH; 25 μg/dose) and CpG ODN 1826 (5'-TCCATGACGTTCCTGACGTT-3'; Coley Pharmaceutical, Wellesley, MA; 50 μg/dose). The amino acid sequence of the irrelevant peptide used in ELISPOT was EGSRNQDWL (Dalton Chemical Laboratories Inc.).

### Tumor challenge

B16-F10 cells (10^4 ^cells/mouse) were implanted subcutaneously in the left flank of C57BL/6 mice which were 6–8 weeks of age at time of challenge. Tumor were measured with calipers every 2–5 days. Data was reported as a percentage of tumor-bearing mice.

### Therapeutic immunization

**Four to **six days after implantation of melanoma cells, mice (5 mice/group) received a single subcutaneous (s.c.) injection of VM containing TRP2: 180–188, TRP2: 181–188, or modified p53: 232–240 (25 μg of peptide/mouse). In addition, mice were injected with vaccine containing mixtures of two peptides (25 μg of each peptide/mouse). The mixtures contained either TRP2:180–188 and modified p53 or TRP2 181–188 and modified p53. VM formulations contained PADRE (25 μg/mouse) and CpG (50 μg/mouse) as adjuvants. The peptides and adjuvants were encapsulated in VM as previously described [1]. In brief, lecithin and cholesterol in a ratio of 10:1 (0.2 g lecithin and 0.02 g cholesterol/dose) were dissolved in chloroform/methanol (1:1;v/v) and the solution was filter-sterilized using a PTFE 0.2 μm filter. Chloroform and methanol were removed under reduced pressure using a rotary evaporator and traces of the solvents were removed from the resulting thin lipid layer *in vacuo*. For liposome encapsulation, peptides with CpG and PADRE were dissolved in sterile PBS and the resulting solution added to the thin lipid layer with mixing to form liposomes. The resulting suspension of liposomes was emulsified in Montanide ISA51 (Seppic, France) by adding the liposome/PBS suspension to ISA51 to form a water-in-oil emulsion (PBS:ISA51; 1:1, v/v; 100 μl/dose). Control mice were injected with either vaccines that contained all components of test vaccines except liposomes or phosphate buffered saline (PBS, 100 μL/injection).

### *Ex vivo *analysis of antigen-specific T cells by ELISPOT

Activated antigen-specific splenocytes harvested from immunized C57BL/6 mice were detected using the BD ELISPOT kit following the manufacturer's instructions (BD Bioscience, San Diego, CA). Briefly, on day 8 post-immunization, a 96-well nitrocellulose plate was coated overnight at 4°C with capture antibody (anti-mouse IFN-γ) and then blocked with complete media. Splenocytes were added to wells at an initial concentration of 1 million cells/well in a volume of 100 μl. Cells in a dilution series were either non-stimulated or stimulated with relevant or irrelevant peptides (10 μg/ml).

PMA (5 ng/ml, Sigma) and ionomycin (500 ng/ml, Sigma), served as positive controls and irrelevant peptide and media alone served as negative controls. The plate was incubated overnight at 37°C/5% CO_2_. The plate was then incubated with detection antibody (a biotinylated anti-mouse IFN-γ antibody), for 2 hours at room temperature. Unbound detection antibody was removed by washing and the enzyme conjugate (Streptavidin-HRP) was added. Following 1 hour incubation at room temperature, unbound enzyme conjugate was removed by washing and the plate was stained with an AEC substrate solution for 20 minutes. The plate was washed, allowed to air dry overnight, and foci of staining were counted using a magnifying lens.

### CD4^+ ^T cell responses to PADRE

The CD4^+ ^T-cell helper response to PADRE was measured by ELISPOT. Eight days post-immunization, splenocytes were recovered from immunized mice and stimulated with PADRE (10 μg/ml). Production of IFN-γ was measured as described above.

### Statistical analysis

Data are expressed as the mean ± SD and statistical significance was determined using paired two-tailed Student t-test (p < 0.05).

## Results

### Eradication of B16-F10 melanoma tumors

**Figure 1:**
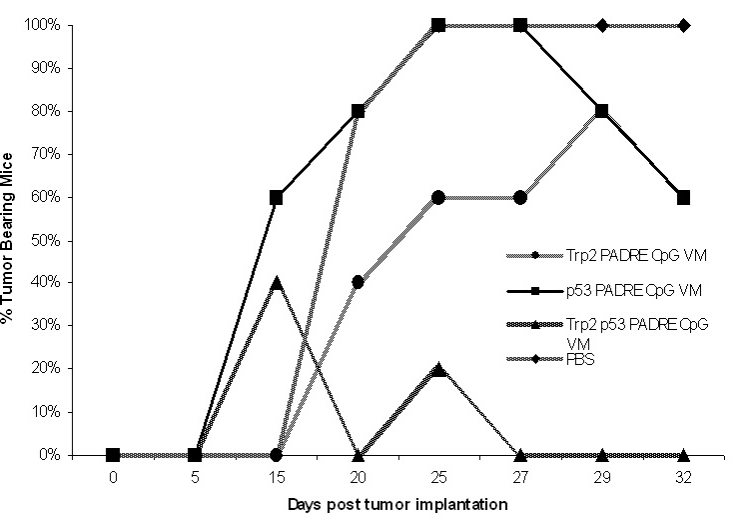
All mice that received PBS (crosses) developed tumors by day 14 post-immunization. A single immunization against a mixture of TRP2:181–188 and modified p53:232–240 in VM (triangles) rendered all mice tumor-free by 21 days post-immunization. A single administration of TRP2:181–188 alone alone (diamonds) in VM rendered 2/5 mice tumor-free by day 16 post-immunization. A single administration of modified p53:232–240 in VM (squares) delayed tumor development in one mouse, but by day 21 post-immunization, all mice in this group were tumor bearing. In the interval between 25 and 27 days post-immunization, two mice in this group became tumor-free.

The B16-F10 melanoma model was used to demonstrate the therapeutic value of VM formulated with tumor-associated self antigens. Based on our observations that eradication of HPV TC1/A2 tumors was more effective when more than one CTL epitope-containing peptide was incorporated in VM [2], a mixture of 2 peptides formulated in a vaccine enhancement platform was used to eradicate B16-F10 melanoma tumors. All mice immunized against a mixture of TRP2:181–188 and modified p53: 232–240 were tumor-free by 21 days post-immunization (Fig. 1). Mice remained tumor-free until day 30–35, after which tumors regenerated in a number of mice. In this representative experiment, vaccinated mice remained tumor-free until day 33. Thereafter, tumors regenerated in two mice by day 35, in a third mouse by day 42 and in a fourth mouse by day 50 post-immunization. In two similar trials, immunization against a mixture of TRP2:180–188 and modified p53:232–240 in VM resulted in tumor rejection in 60–80% of treated mice within 26–32 days post-immunization, (data not shown) and these mice remained tumor-free at the end of the trial (day 40 post-immunization). In contrast, when TRP2 and p53 epitopes were combined with CpG and ISA51 (control vaccination, no liposomes), no more than 20% of mice remained tumor-free mice until day 25–30 and tumors regenerated in all vaccinated mice (data not shown).

Immunization against TRP2:180–188 alone plus CpG in VM eradicated tumors in 40% of the mice by 16 days post-immunization. These mice remained tumor-free for the entire monitoring period. Immunization against TRP2:181–188 alone also suppressed tumors in 40% of mice by 16 days post-immunization, but tumors regenerated in these mice by day 30 post-immunization (data not shown).

Immunization of mice against modified p53:232–240 alone delayed tumor development in one mouse but by day 21 post-immunization all mice were bearing tumors. Twenty percent of mice became tumor-free in the interval between day 25 and 27 post-immunization. All control mice injected with PBS developed tumors by day 14 post-immunization.

### Induction of cytotoxic T cells

**Figure 2:**
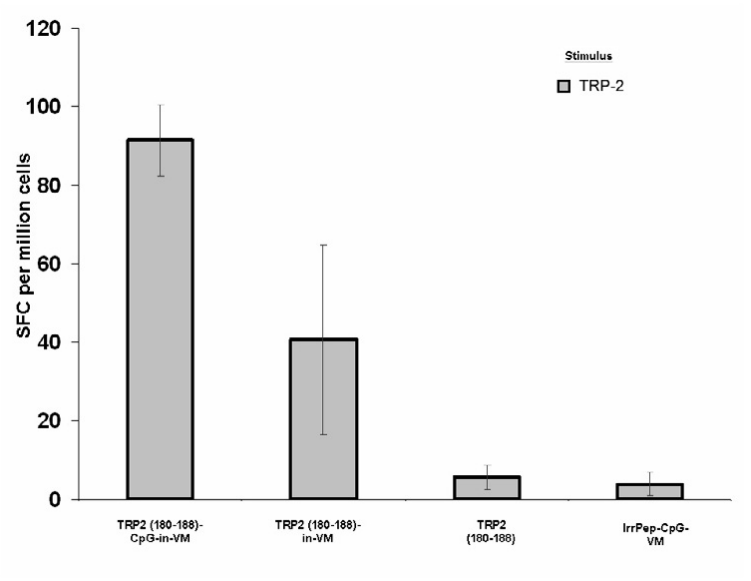
Ex-vivo detection of modified TRP2:180–188-specific IFN-γ producing splenocytes (spot forming cells, SFC) in mice 8 days following a single immunization against TRP2:180–188 in VM. Mice immunized against TRP2:180–188 in VM with CpG adjuvant produced the largest number of cytotoxic T cells. Omission of CpG from the vaccine produced approximately one-half as many SFC. TRP2:180–188 alone and an irrelevant peptide with CpG in VM produced background numbers of SFC.

Previous reports have suggested that TRP2, and in particular TRP2 epitope 180–188, are poorly immunogenic [3,24-34]. In these studies, immunization against TRP2:180–188 required multiple administrations with CpG ODN to induce CTL responses. Even when complemented by PADRE and in IFA, this composition did not result in protection against B16-F10 tumor growth [25,28]. To assess the ability of VM to enhance the immune response to TRP2:180–188, mice (3–5/group) were immunized against these peptides or an irrelevant peptide in VM. Splenocytes from individual mice were harvested 8 days post-immunization and stimulated *in vitro *for 12 hours with TRP2:180–188. The number of TRP2:180–188-activated IFN-γ-producing T cells was determined by ELISPOT assay. A significant increase in the number of TRP2:180–188-specific IFN-γ-producing cells occurred by 8 days post-immunization (Fig. 2). This experiment indicated that VM without adjuvant could induce a CTL response against TRP2:180–188. The presence of CpG ODN in VM increased the CTL response against the epitope by approximately two-fold. TRP2:180–188 alone produced a background CTL response as did immunization against an irrelevant peptide. Similar results were obtained using splenocytes from mice vaccinated with TRP2:181–188 instead of TRP2:180–188 (data not shown). The CTL response to TRP2:181–188 with CpG ODN but without VM was approximately 20% of the CTL response to TRP2:181–188 with CpG ODN in VM. These results indicate that VM stimulates a robust CTL response to TRP2:180–188 and TRP2:181–188.

**Figure 3:**
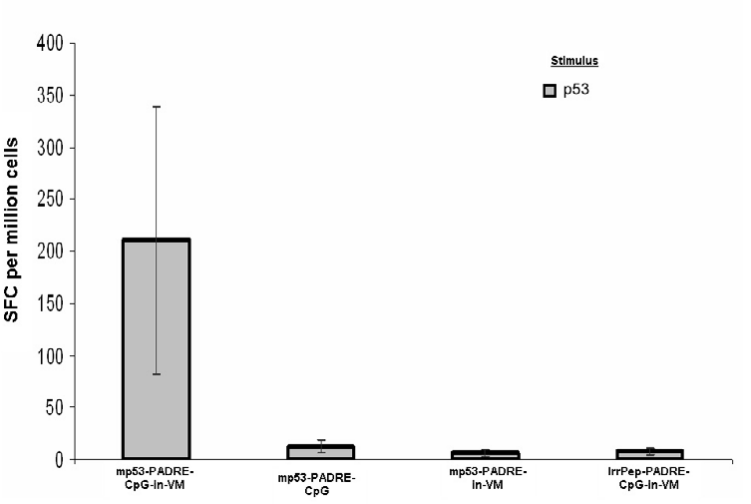
Ex-vivo detection of modified p53:232–240-specific IFN-γ producing splenocytes (spot forming cells, SFC) in mice 8 days following a single immunization against modified p53:232–240 in VM. Mice immunized against modified p53:232–240 peptide in VM with CpG and PADRE adjuvants produced the largest number of cytotoxic T cells. Without VM or omission of CpG from the VM formulation produced low numbers of modified p53:232–240-specific IFN-γ producing splenocytes. Vaccination against an irrelevant peptide (IrrPep) also produced low numbers of IFN-γ producing splenocytes.

To assess the ability of VM to enhance the immune response to modified p53:232–240, mice (3–5/group) were immunized against modified p53:232–240 in VM, in VM without CpG, without VM liposomes, or against an irrelevant peptide in VM. Splenocytes from individual mice were harvested 8 days post-immunization and stimulated *in vitro *with modified p53:232–240. A significant increase in the number of modified p53:232–240-specific IFN-γ-producing cells was observed in mice immunized against modified p53:232–240 in VM by 8 days post-immunization (Fig. 3). This vaccine formulation without CpG produced background numbers of modified p53:232–240-specific splenocytes as did formulations that lacked the liposome component of VM (modified p53:232–240-PADRE-CpG and modified p53:232–240-PADRE). Immunization against an irrelevant peptide also produced similar background numbers of modified p53:232–240-specific splenocytes.

**Figure 4:**
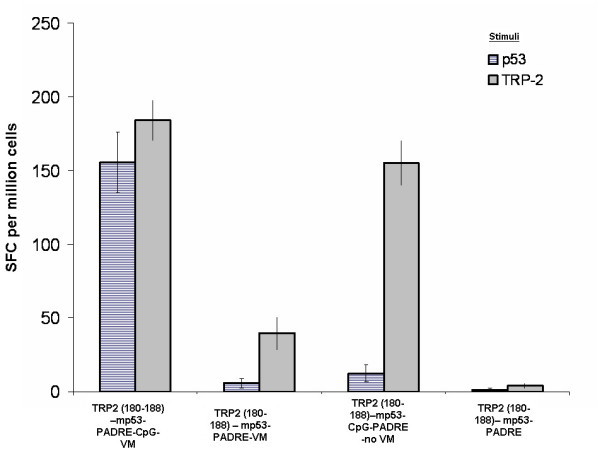
Spleens from mice immunized against a mixture of TRP2:180–188 and modified p53:232–240 in VM contained high numbers of TRP2:180–188-specific (right bar) and modified p53:232–240-specific (left bar) IFN-γ producing cells. In contrast, spleens from mice immunized against a mixture of TRP2:180–188 and modified p53:232–240 without VM contained high numbers of TRP2:180–188-specific IFN-γ producing cells but low numbers of modified p53:232–240-specific IFN-γ producing cells. The spleens of mice immunized with VM-formulated vaccine without CpG ODN had low numbers of both TRP2:180–188- and modified p53:232–240-specific IFN-γ producing cells as did the spleens of mice from mice immunized without VM.

Melanoma cells express more than one tumor-associated protein simultaneously. TRP2 is a tumor-associated protein limited to melanoma, whereas, p53 is over-expressed in a wide variety of cancers. For example, the p53 gene is commonly mutated in lung, colon, and breast cancers. The observation that immunization against a mixture of peptides derived from TRP2 and p53 was more effective in eradicating B16-F10 melanoma tumors than immunization against each of these peptides alone suggests that use of VM induces a CTL response against more than one CTL epitope simultaneously. To evaluate the ability of VM to raise a simultaneous immune response against two targets, mice (3/group) were immunized once against a mixture of TRP2:180–188 and modified p53:232–240 peptides, with and without VM, or with and without CpG ODN (Fig. 4). All vaccine formulations contained water-in-oil emulsions. Formulations without VM contained all vaccine ingredients except liposomes. Spleens were collected 8 days post-immunization and the number of IFN-γ producing cells was measured by ELISPOT assay. A single immunization with VM containing both TRP2:180–188 and modified p53:232–240 antigens stimulated a robust IFN-γ response against both antigens simultaneously. Spleens from mice immunized with VM contained similar numbers of TRP2:180–188-specific and modified p53:232–240-specific IFN-γ producing cells. In contrast, spleens from mice immunized by a single vaccination of a mixture of TRP2:180–188 and modified p53:232–240 without VM produced a robust TRP2:180–188-specific response but a weak modified p53:232–240-specific response. Vaccination against a mixture of TRP2:180–188 and modified p53:232–240 without CpG resulted in a poor TRP2:180–188-specific IFN-γ response and a slightly better modified p53:232–240-specific response. Without VM, both TRP2:180–188- and modified p53:232–240-specific IFN-γ responses were poor.

### CD4^+ ^T cell responses to PADRE

**Figure 5:**
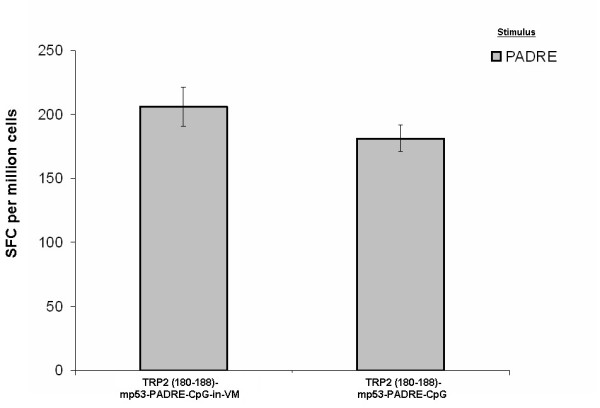
Ex-vivo detection of PADRE-specific IFN-γ producing splenocytes (spot forming cells, SFC) in mice (N = 5) 8 days following a single immunization against a mixture of TRP2:180–188 and modified p53:232–240 with and without VM.

CD4^+ ^T-cell help is essential for the differentiation and expansion of CTLs [35], as well as their maturation into functional memory CTLs [36]. To achieve CD4^+ ^T-cell help, a universal T helper epitope, PADRE was used in VM [37]. A strong PADRE-specific CD4+ T cell help was induced in mice immunized against TRP2:180–188 with and without VM (Fig. 5). These results along with figures 1 and 2 show the simultaneous induction of immune responses to multiple MHC class I and II peptides when encapsulated in VM.

## Discussion

There is an unmet need for new vaccines that are cost effective, safe and yet mount robust, effective and durable immune responses [38]. Liposomes have been described as safe vaccine adjuvants but their use has been limited to aqueous carriers, such as saline, phosphate buffered saline or oil-in-water emulsions. In contrast, liposomes stabilized to remain intact in hydrophobic carriers or water-in-oil emulsions (VM) significantly enhance both humoral and CTL immune responses [1,2,39]. To enhance immunity, the VM platform requires both liposomes and a hydrophobic carrier. This combination allows excellent antigen delivery and presentation to the immune system resulting in a durable immune response following a single vaccination. Most vaccines studied using the same mouse model as employed in our studies, are only useful as prophylactic vaccines. Clearance of established tumors by immunization against peptide antigens using VM have also been demonstrated in two independent HPV-cervical cancer models [1,2]. This strategy is particularly effective when treating virally induced cancers. Self-antigens are tightly guarded by tolerance mechanisms and consequently, tumors presenting "self" antigens are more difficult to treat. Therefore, an effective therapeutic cancer vaccine platform with widespread applications must have the ability to induce immune responses against tumor-associated self antigens. Consequently, a therapeutic vaccine against melanoma must activate and expand T cell clonotypes that have escaped thymus clonal deletion but nevertheless are capable of targeting self epitopes on the surface of tumors.

The B16-F10 melanoma tumor model in C57BL/6 mice has been used in many pre-clinical melanoma studies [3,24-34]. Consequently, this model was chosen to test VM-based melanoma vaccines that employ "self" antigens such as TRP2 and p53. Although p53 is rarely used as antigen for melanoma immunotherapy, an increased content of p53 in melanoma cell lines and induction of p53-specific CTL responses have been reported by others [4,5,10,20,23]. Cell-mediated immune responses to TRP epitopes correlated positively with tumor regression [12,16,40,41]. Immune responses against these antigens are difficult to achieve because of immune tolerance mechanisms [42]. Various approaches have been adopted for the generation of CTL responses against B16, including the use of antigen-pulsed DCs or transfection of B16 cells with the gene for GM-CSF [40,43,44]. Recently, Jerome and colleagues demonstrated high levels of CTL responses upon vaccination with TRP2 and CpG in liposome [3].

Surprisingly, preliminary studies employing VM detected an *ex vivo *CTL response against TRP2:180–188 in the absence of adjuvants (Fig. 2). Addition of CpG adjuvant however significantly increased the number of TRP2:180–188-specific IFN-γ producing splenocytes. To increase the number of IFN-γ producing splenocytes that were specific for modified p53:232–240, the presence of both PADRE and CpG were required. Since studies aimed at developing a therapeutic HPV vaccine demonstrated that multiple peptide antigens result in a better outcome than a single peptide antigen [2], a mixture of TRP2:180–188 and modified p53:232–240 in VM was evaluated to determine if T cell activation would be directed against one or both peptides. The TRP2:180–188-, modified p53:232–240- and PADRE-specific IFN-γ producing splenocytes were expanded in mice immunized with the mixture of three epitopes in VM (Figs 4 and 5). Without VM, only the number of TRP2:180–188-specific IFN-γ producing splenocytes was above background suggesting immuno-dominancy favoring TRP2-specific responses. Although a T helper response was mounted when all three epitopes plus CpG were used without VM, such helper response only supported CTL responses to one of the peptides (TRP2:180–188) and resulted in tumor suppression until day 25–30 in 20–40% of treated mice.

As a realistic model for melanoma immunotherapy, we used peptide antigens derived from the naturally expressed tumor-associated antigens TRP2 and p53 in the B16-F10 melanoma model. Poor immunogenicity of peptides and the fact that B16-F10 cells express very low amounts of MHC class 1 molecules make the B16-F10 model a challenging model for T-cell based immunotherapy [45]. Immunization with liposome-encapsulated TRP2 peptide which were then mixed with CpG ODN caused complete tumor rejection in only 20% of vaccinated mice [3]; VM, on the other hand, caused tumor rejection in all treated mice when two antigens were targeted. The ability to co-deliver antigens and CpG adjuvant by encapsulation in liposomes is a significant advantage for the VM platform; co-encapsulation of antigen and adjuvant within the same liposome is superior to encapsulation of each in separate liposomes [46]. Our study demonstrates that co-encapsulation of adjuvant with peptide antigens derived from two tumor-associated antigens in VM is superior to use of a single peptide antigen. For example, TRP2:180–188 and modified p53:232–240 encapsulated in VM with PADRE and CpG ODN adjuvants caused complete tumor eradication in 100% of vaccinated mice. The same treatment with only one CTL epitope (TRP2:180–188) caused complete tumor suppression for at least 20 days in 60% of vaccinated mice. VM-based vaccines containing more than two peptide antigens may improve the outcome of therapeutic melanoma vaccines as a result of VM's ability to overcome immuno-dominancy.

In summary, the VM vaccine platform simultaneously induces a robust CTL response to more than one peptide antigen contained therein. Immunization against two CTL epitopes in VM formulated with a T helper peptide and CpG adjuvant eradicated tumors in all treated mice. Although regeneration of melanoma tumors occurred in a significant number of mice that had been rendered tumor-free by vaccination, the strong CTL responses to multiple peptides from self-antigens by a single immunization and the rejection of aggressive 6-day old B16-F10 tumors to our knowledge have never been reported. Targeting more than 2 tumor-associated antigens in VM may improve the efficacy of the vaccine and reduce melanoma tumor regeneration.

## Conclusion

VacciMax^®^, a liposome/Water-in-oil based vaccine delivery system, enhances the immunogenicity of melanoma-associated CTL epitopes simultaneously. A single administration of such vaccine elevated specific CTL responses and resulted in the rejection of 6-day old B16-F10 tumors.

## Abbreviations

CTL, cytotoxic T lymphocyte; ELISPOT, enzyme-linked immunosorbent spot; TRP-2, Tyrosinase-Related Protein-2; PADRE, pan DR epitope; VM, VacciMax^®^.

## Competing interests

MM, AFO, EKS, GMW and PD are employed by ImmunoVaccine Technologies Inc., the holder of patents relating to the use of liposomes in oil emulsions in vaccine applications as described in this manuscript. However, all experimental data in the current study were evaluated independently by other authors and researchers who have no financial interests in the content of this manuscript.

## Authors' contributions

MM contributed largely in experimental design, coordination of experiments and analysis of results. BP contributed in designing the vaccines and reviewing manuscript. MK helped technically and critically reviewed the paper. AFO, EKS, and GMW performed all vaccine preparation, ELISPOT, cell culture, tumor implantation and tumor measurements. RGB, participated in writing the paper. PD conceived the study, designed the vaccine combination, participated in writing the manuscript and analysis of results. All authors have read and approved the final manuscript.
